# The integrated stress response kinase GCN2 prevents ZAKα-Dependent inflammatory hyperactivation in macrophages

**DOI:** 10.1016/j.jbc.2026.113452

**Published:** 2026-08-14

**Authors:** Rodrigo Dias Requião, Lincon Felipe Lima-Silva, Paulo Estevão, Webster Leonardo Guimarães Costa, Nicholas Camargo Zani, Israel Costa Vasconcelos, Ítalo Esposti Poly Silva, Teng Teng Xu, Heloísa Monteiro do Amaral Prado, Barbara Steigenberger, Mario Henrique Bengtson, Peter J. Murray, Pedro M. Moraes-Vieira

**Affiliations:** 1 Department of Genetics, Evolution, Microbiology and Immunology, Institute of Biology (IB), University of Campinas (UNICAMP), Campinas, Brazil; 2 Department of Immunoregulation, Max Planck Institute of Biochemistry (MPIB), Max Planck Society (MPG), Martinsried, Germany; 3 Department of Biochemistry and Tissue Biology, Institute of Biology (IB), University of Campinas (UNICAMP), Campinas, Brazil; 4 Center for Medicinal Chemistry (CQMED), Universidade Estadual de Campinas (UNICAMP), Campinas, Brazil; 5 Max Planck Institute of Biochemistry Core Facility, Max Planck Society (MPG), Martinsried, Germany; 6 Experimental Medicine Research Cluster (EMCR), University of Campinas (UNICAMP), Campinas, Brazil

**Keywords:** GCN2 signaling, inflammation regulation, macrophage activation, translation control, ZAKα pathway

## Abstract

Macrophages orchestrate inflammation through rapid and extensive proteome remodeling, yet the translational programs governing macrophage activation remain poorly defined. Here, we show that classically activated macrophages (lipopolysaccharide + interferon-γ-treated) and alternatively activated macrophages (IL-4-treated) engage fundamentally distinct translational trajectories. Whereas alternatively activated macrophages sustain elevated protein synthesis, classically activated macrophages undergo a rapid but transient increase in translation that is subsequently restrained by the integrated stress response kinase General Control Nonderepressible 2 (GCN2). Using puromycin incorporation and quantitative proteomics, we demonstrate that GCN2-mediated phosphorylation of eukaryotic translation initiation factor 2α limits global translation and constrains the proinflammatory response. Genetic loss of GCN2 results in excessive translation and hyperinflammation driven by the ribosome-associated stress sensor ZAKα (*Map3k20*). Pharmacological inhibition of ZAKα in GCN2-deficient macrophages selectively normalizes tumor necrosis factor α secretion, establishing a functional regulatory axis in which GCN2 suppresses ZAKα-dependent inflammatory signaling. Together, these findings redefine translational control as a central checkpoint in macrophage activation, revealing how GCN2 mitigates ribosomal stress to prevent inflammatory hyperactivation, with potential therapeutic implications for tumor necrosis factor α -driven inflammatory diseases.

Macrophages deploy rapid changes in their proteome to mount inflammatory responses, making translational control a critical yet poorly understood regulatory node in immune regulation. While early studies established that macrophage differentiation and inflammatory activation are associated with increased polysome formation and protein synthesis (1, 2, 3, 4, 5), these works are constrained by key limitations. Existing findings often rely on short observation windows or transformed cell lines, whose proliferative nature obscures general cellular growth. Furthermore, the translational landscape of alternatively activated (*e.g.*, M(IL-4)) macrophages remains incompletely understood.

Critically, the molecular mechanisms that govern these changes in global translation are unknown. Translational rates are primarily determined at the initiation phase, chiefly through two well-defined pathways, the eukaryotic translation initiation factor 2α (eIF2α) and 4EBP1/2 axes (6). Phosphorylation of eIF2α inhibits formation of the eIF2–GTP–tRNAᵢᴹ^e^ᵗ ternary complex, globally repressing translation initiation (6). Conversely, phosphorylation of 4EBP1/2 releases its inhibition of eIF4E, promoting cap-dependent translation (6). Whether and how these central regulatory pathways are engaged to shape the distinct functional responses of classically *versus* alternatively activated macrophages remains a fundamental unanswered question.

Here, we systematically define the translational dynamics and their regulatory mechanisms in primary macrophages undergoing inflammatory or reparative activation. We reveal that macrophages stimulated with lipopolysaccharide (LPS) plus interferon-γ (IFNγ) or interleukin 4 (IL-4) engage strikingly distinct translational programs that are differentially coupled to the eIF2α pathway. Focusing on inflammatory macrophages, we identify the stress-sensing kinase General Control Nonderepressible 2 (GCN2) as a central regulator that restrains excessive protein synthesis during activation. Importantly, loss of GCN2 leads to exaggerated inflammatory responses, indicating that translational control is not merely adaptive but essential to prevent hyperinflammation.

Beyond its established role in amino acid sensing, GCN2 is increasingly recognized as a broader integrator of translational stress (6, 7). In parallel, the MAP3K ZAKα has also been implicated in signaling pathways that respond to disturbances in ribosomal function (8, 9). While GCN2 and ZAKα are typically studied in distinct contexts, both are activated under conditions of ribosomal or translational stress, suggesting that they may operate as coordinated checkpoints during immune activation. However, whether and how these pathways intersect in macrophages has remained unknown.

Together, our study identifies a previously unappreciated regulatory axis in which GCN2 constrains inflammatory signaling by limiting the engagement of ZAKα-dependent pathways, thereby preventing excessive TNFα production. These findings establish translational stress surveillance as a critical mechanism for tuning inflammatory intensity during macrophage activation.

## Results

### Inflammatory and reparative macrophages exhibit divergent translational dynamics

**Figure 1:**
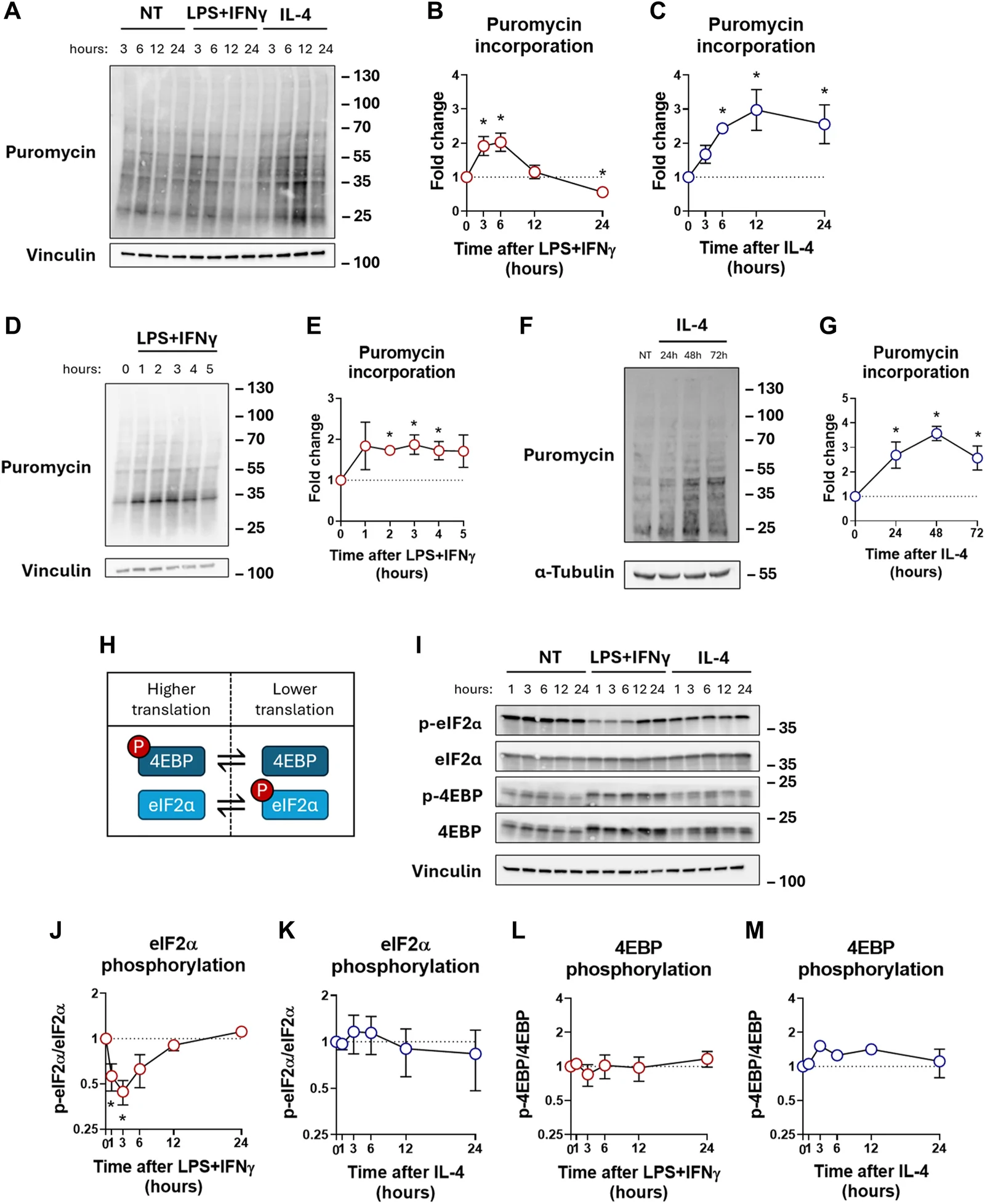
**Translation modulation in activated macrophages.***A*, puromycin incorporation assay measuring nascent protein synthesis in BMDMs treated with LPS + IFNγ or IL-4 over time compared with NT. *B and C,* quantification of puromycin incorporation shown in *(A)* after stimulation with LPS + IFNγ (n = 4) or IL-4 (n = 3), illustrating their opposing effects on global translation rates. *D and E,* puromycin incorporation in BMDMs stimulated with LPS + IFNγ for 1–5h (n = 3). *F and G,* puromycin incorporation in BMDMs stimulated with IL-4 for 24–72h (n = 3). *H,* schematic representation illustrating how phosphorylation of eIF2α and 4EBP regulates translation initiation by modulating ribosome recruitment and initiation complex assembly. *I,* immunoblot analysis of eIF2α and 4EBP phosphorylation in BMDMs treated with LPS + IFNγ or IL-4 over time compared with NT, showing activation of canonical translational control pathways in response to different activations. *J and K*, quantification of eIF2α phosphorylation from *(I)* after stimulation with LPS + IFNγ (n = 3) or IL-4 (n = 3), indicating differential modulation pathway activation under different stimuli. *L and M,* quantification of 4EBP phosphorylation from *(I)* after stimulation with LPS + IFNγ (n = 3) or IL-4 (n = 3), indicating no modulation of cap-dependent translation. Data are presented as mean ± SEM. Statistical significance was determined by one-way ANOVA with Dunnett’s multiple comparisons test. ∗ = *p* < 0.05. BMDM, bone marrow–derived macrophage; eIF2α, eukaryotic translation initiation factor 2α; IFNγ, interferon-γ; LPS, lipopolysaccharide; NT, non-treated.

To define how translational control contributes to macrophage polarization, we measured global protein synthesis in bone marrow–derived macrophages (BMDMs) stimulated toward inflammatory (LPS + IFNγ) or reparative (IL-4) states. Using puromycin incorporation, we observed strikingly distinct temporal patterns of protein synthesis. In LPS + IFNγ activated macrophages, M(LPS + IFNγ), translation rates surged rapidly within the first hours following stimulation, reaching a peak between 3 and 6 h, before sharply declining by 24 h to levels below those observed in unstimulated cells (Fig. 1, *A* and *B*). This biphasic response, an initial burst followed by repression, contrasts with prior reports that described increased translation without documenting a subsequent decline (1, 2, 3, 4, 5). Importantly, this decrease in translation was not due to loss of cell viability, which remained high at 24 h (Fig. S1). A high-resolution time course confirmed the rapid onset of increased translation, which began within the first hour and was sustained at elevated levels throughout the first 5 h of activation (Fig. 1, *D* and *E*).

In contrast, IL-4 treated macrophages, M(IL-4), exhibited a sustained translational program. Translation rates increased following stimulation and remained elevated after 24 h (Fig. 1, *A* and *C*). This sustained activity persisted for up to 72 h, with only a gradual decrease from peak levels, and remained significantly higher than unstimulated controls throughout this period (Fig. 1, *F* and *G*). Thus, proinflammatory and reparative activation drive fundamentally distinct translational trajectories, with M(LPS + IFNγ) macrophages exhibiting a transient, self-limiting response, whereas M(IL-4) macrophages maintain prolonged translational activity.

Having established these distinct translational dynamics, we next sought to define the regulatory mechanisms governing translation initiation. We focused on two major regulatory nodes: eIF2α, whose phosphorylation suppresses translation initiation, and 4EBP, whose phosphorylation promotes cap-dependent translation (Fig. 1*H*). In M(LPS + IFNγ) macrophages, the early surge in translation coincided with a marked decrease in eIF2α phosphorylation, consistent with a transient release of translational repression. Additionally, the subsequent decline in translation at later time points coincided with restoration of eIF2α phosphorylation (Fig. 1, *I* and *J*). In contrast, phosphorylation of 4EBP did not correlate with translation rates, ruling it out as the primary driver of this response (Fig. 1, *I* and *L*). This specific engagement of the eIF2α axis was not observed in M(IL-4) macrophages, where neither eIF2α nor 4EBP phosphorylation patterns aligned with sustained translation, indicating that reparative macrophages rely on distinct translational control mechanisms (Fig. 1, *I*, *K* and *M*).

### GCN2 is the dominant eIF2α kinase restricting early inflammatory translation

**Figure 2:**
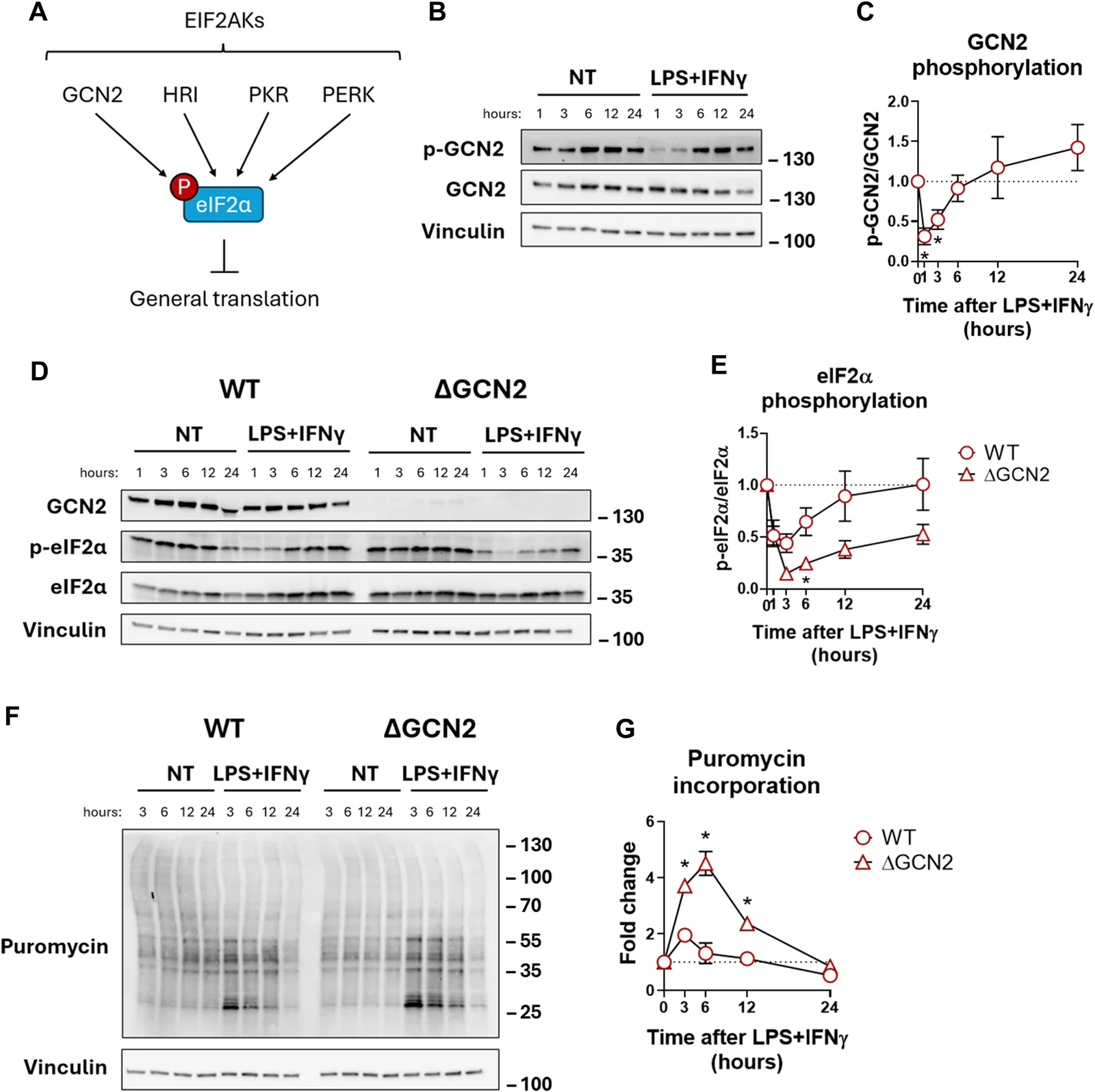
**Role of GCN2 in translational control in M(LPS + IFNγ) macrophages.***A,* simplified schematic representation of EIF2α kinases (EIF2AKs) regulating translation in response to cellular stress. *B* and *C*, immunoblot analysis of GCN2 phosphorylation in BMDMs treated with LPS + IFNγ over time compared with NT, showing activation kinetics of this kinase (n = 3). *D and E,* immunoblot analysis of eIF2α phosphorylation in LPS + IFNγ-treated BMDMs over time in WT and ΔGCN2 cells, revealing the contribution of GCN2 to eIF2α phosphorylation (n = 3). *F and G*, puromycin incorporation assay in LPS + IFNγ-treated BMDMs over time in WT and ΔGCN2 cells compared with NT, assessing global translation (n = 3). Data are presented as mean ± SEM. Statistical significance was determined by one-way ANOVA with Dunnett’s multiple comparisons test for *(C)* and two-way ANOVA with Turkey’s multiple comparisons test for *(E)* and *(G)*. ∗ = *p* < 0.05. BMDM, bone marrow–derived macrophage; eIF2α, eukaryotic translation initiation factor 2α; GCN2, General Control Nonderepressible 2; IFNγ, interferon-γ; LPS, lipopolysaccharide; NT, non-treated.

Next, we sought to determine how phosphorylation of eIF2α is regulated in M(LPS + IFNγ) macrophages. We focused our analysis on inflammatory macrophages, as M(IL-4) macrophages did not exhibit detectable modulation of eIF2α phosphorylation. Phosphorylation of eIF2α is catalyzed by one of four stress-responsive kinases: GCN2, PKR, PERK, or HRI (2, 6) (Fig. 2*A*). We chose to investigate the role of GCN2 based on prior studies implicating this kinase in macrophage inflammatory activation and immune regulation (10, 11, 12).

Analysis of GCN2 phosphorylation revealed a dynamic pattern during LPS + IFNγ stimulation. Phosphorylation of GCN2 decreased during the early hours of activation and increased at later time points, closely mirroring the temporal dynamics of eIF2α phosphorylation and coinciding with the transient surge and subsequent repression of protein synthesis (Fig. 2, *B* and *C*). These observations suggested a regulatory role for GCN2 in controlling translation during inflammatory macrophage activation.

**Figure 3:**
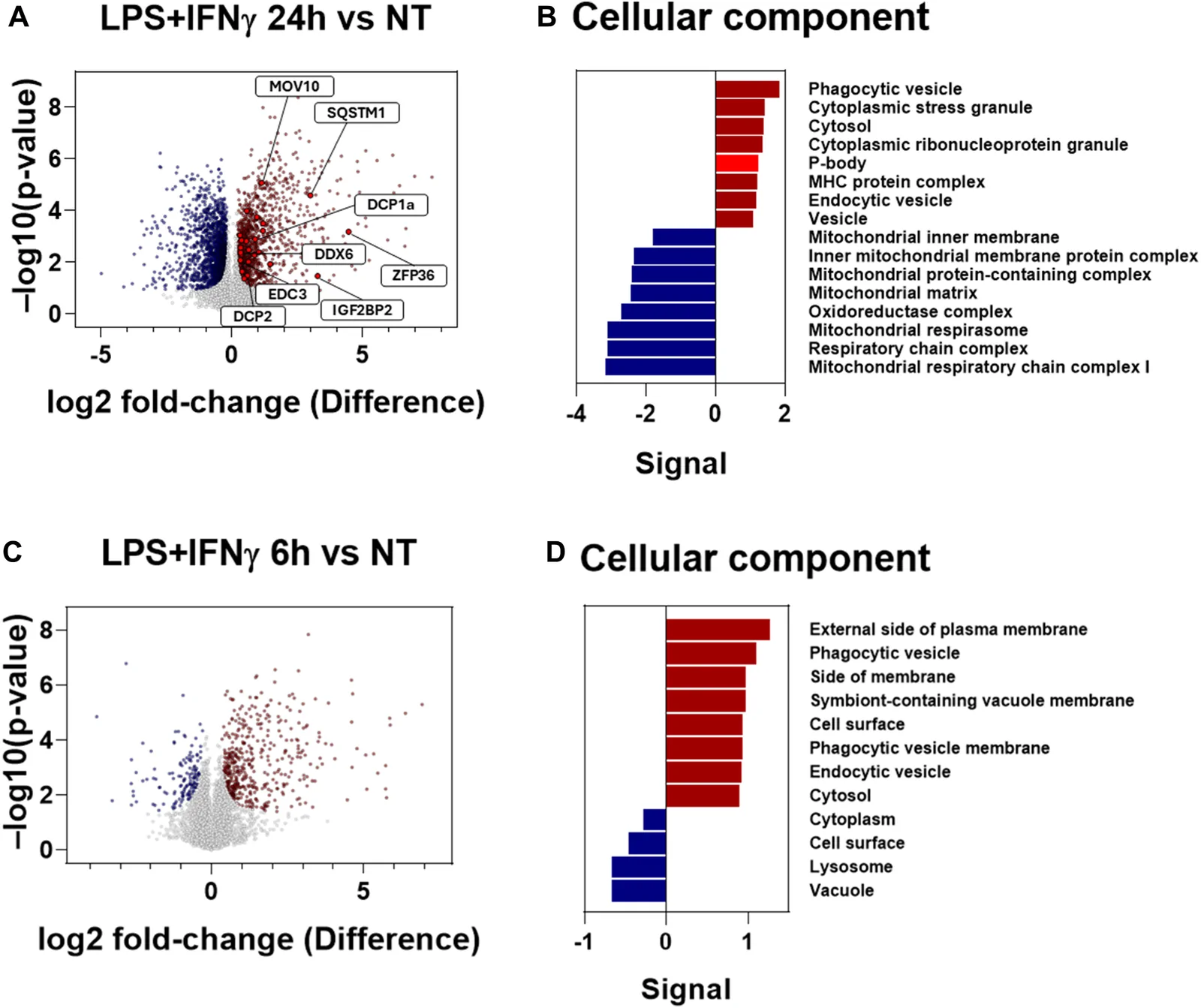
**Proteome analysis of M(LPS + IFNγ) macrophages.***A,* global proteomic profiling of BMDMs stimulated with LPS + IFNγ for 24 h compared with NT controls, highlighting proteins associated with the GO category “P-body.” *B,* GO cellular component enrichment analysis of proteins identified in *(A)*, showing enrichment of P-body–related components (highlighted in *light red*). *C,* global proteomic profiling of BMDMs stimulated with LPS + IFNγ for 6 h *versus* NT, capturing earlier changes in protein abundance. *D,* GO cellular component enrichment analysis of proteins altered in *(C)*. BMDM, bone marrow–derived macrophage; GO, gene ontology; IFNγ, interferon-γ; LPS, lipopolysaccharide; NT, non-treated.

To directly assess the contribution of GCN2 to translational regulation, we examined puromycin incorporation and eIF2α phosphorylation in GCN2-deficient (ΔGCN2) BMDMs generated from B6.129S6-Eif2ak4^tm1.2Dron^/J mice (The Jackson Laboratory) and WT C57BL/6 controls. Loss of GCN2 resulted in markedly reduced eIF2α phosphorylation following LPS + IFNγ stimulation, and this reduction persisted even at 24 h, a time point at which eIF2α phosphorylation is restored to near-baseline levels in WT macrophages (Fig. 3, *D* and *E*). These data indicate that GCN2 is a major determinant of eIF2α phosphorylation in activated inflammatory macrophages.

Consistent with impaired eIF2α-mediated translational repression, ΔGCN2 macrophages exhibited increased rates of puromycin incorporation during the early phase of activation. Notably, despite this early increase, translation rates declined by 24 h to levels comparable to those observed in WT cells (Fig. 3, *F* and *G*), suggesting that additional, GCN2-independent mechanisms contribute to late-phase translational repression. Together, these results identify GCN2 as the dominant early brake on protein synthesis in inflammatory macrophages, functioning through eIF2α phosphorylation to maintain translational homeostasis during acute inflammatory activation.

### Late inflammatory activation is associated with translational stress

The decline in protein synthesis and the concomitant upregulation of GCN2 activity observed at 24 h in M(LPS + IFNγ) macrophages suggested the induction of a translational stress response. To directly assess this possibility, we performed a comparative proteomic analysis at 6 h, corresponding to peak translational activity, and at 24 h, when global translation is markedly reduced.

Gene Ontology (GO) analysis of enriched cellular compartment pathways at 24 h revealed the expected induction of inflammatory and phagocytosis-related processes, including “Phagocytic vesicle,” “Endocytic vesicle,” and “MHC protein complex” pathways. Notably, this analysis also identified significant enrichment of “Cytoplasmic ribonucleoprotein granule” and “P-body” categories (Fig. 3, *A* and *B*), both of which are canonical hallmarks of translational repression and cellular stress responses (13).

In contrast, these stress-associated pathways were entirely absent from the 6-h proteome, indicating that translational stress emerges specifically during the late phase of inflammatory activation (Fig. 3, *C* and *D*). Direct comparison of the 24-h and 6-h proteomes further underscored a functional shift between these time points, characterized by enhanced representation of phagocytic and antigen presentation pathways alongside a coordinated reduction in proteins associated with mitochondrial oxidative phosphorylation (Fig. S2).

Together, these data demonstrate that M(LPS + IFNγ) macrophages undergo a late-phase translational stress program, temporally coincident with translational repression and increased GCN2 activity, suggesting engagement of adaptive stress response pathways during prolonged inflammatory activation.

### Translational elongation perturbation enhances inflammatory cytokine expression

GCN2 is activated by amino acid starvation (7) and by translational elongation stress associated with ribosome stalling (8, 9). To investigate whether amino acid depletion might underlie GCN2/eIF2α activation in activated macrophages, we compared eIF2α phosphorylation in cell cultures in which media was refreshed every 3 h following stimulation, thereby replenishing extracellular amino acid availability, to cultures maintained in unchanged media. We did not observe differences in eIF2α phosphorylation between these conditions, suggesting that bulk amino acid depletion is unlikely to account for GCN2/eIF2α activation in M(LPS + IFNγ) macrophages (Fig. S3, *A* and *B*).

To assess whether canonical ribosome collision–associated quality control pathways were engaged following macrophage activation, we examined ubiquitination of the ribosomal protein RPS10, a classical marker of ribosomal stress (14, 15, 16). Under our experimental conditions, we did not detect robust RPS10 ubiquitination in response to either inflammatory stimulation (Fig. S3*C*) or canonical anisomycin (ANS) treatment (Fig. S3*D*), indicating that this readout is not readily detectable in primary macrophages in our system.

**Figure 4:**
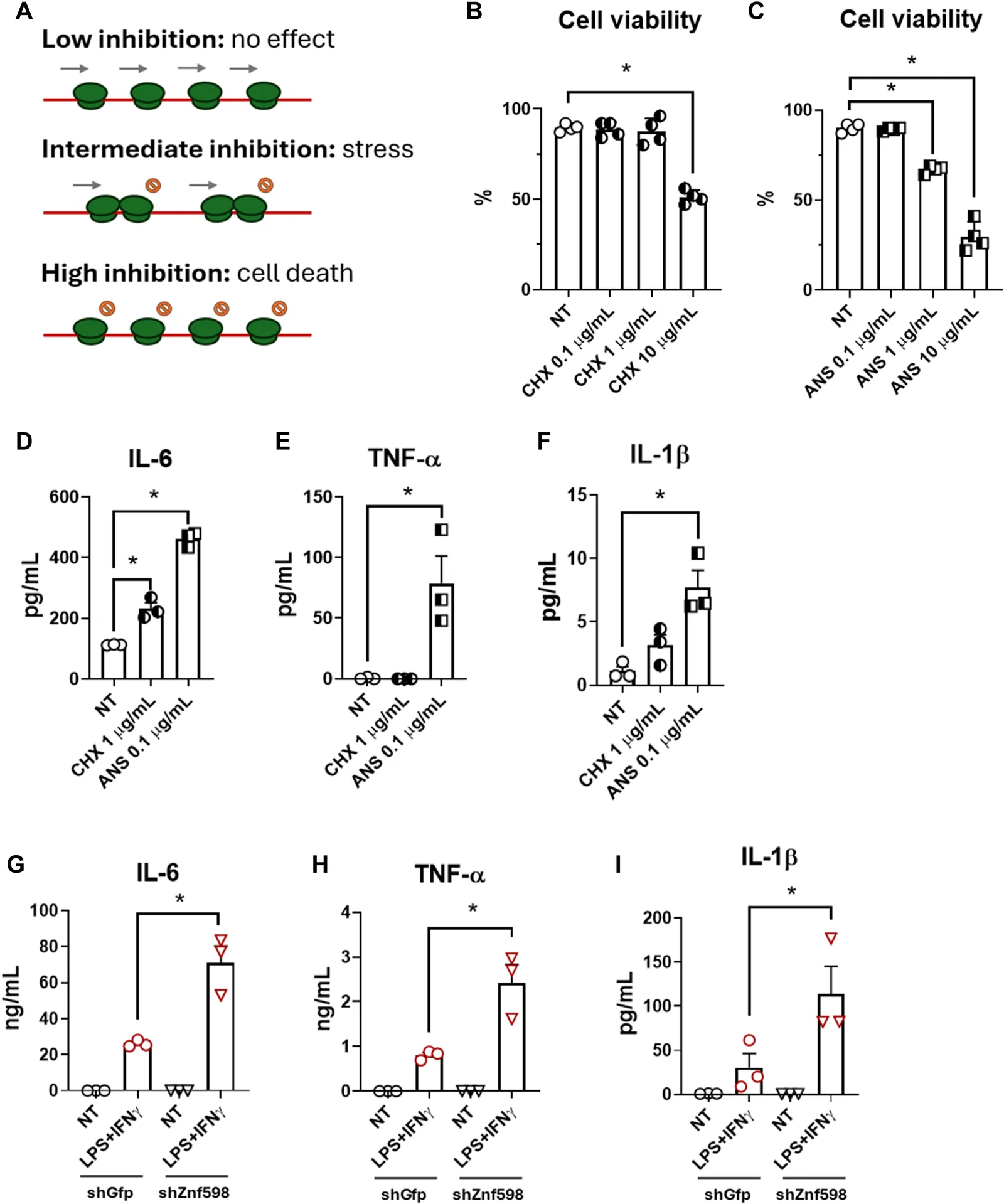
**Effects of translational stress and ribosome quality control on proinflammatory activation in BMDMs.***A,* schematic model depicting the consequences of translation inhibition: low inhibition has minimal effect; intermediate inhibition induces translational stress, and high inhibition triggers cell death. *B and C,* cell viability measured after treatment with increasing concentrations of translational inhibitors cycloheximide and anisomycin (n = 4). *D–F,* cytokine expression measured 24 h after treatment with cycloheximide or anisomycin, demonstrating that translational stress is sufficient for proinflammatory activation (n = 3). *G–I,* cytokine expression after 24 h of LPS + IFNγ stimulation, with or without lentiviral shRNA–mediated knockdown of ZNF598, showing that impairment of ribosome quality control amplifies the inflammatory response (n = 3). Data are presented as mean ± SEM. Statistical significance was determined by one-way ANOVA with Dunnett’s multiple comparisons test for *(B–F)* and two-way ANOVA with Tukey’s multiple comparisons test for *(G–I)*. ∗ = *p* < 0.05. BMDM, bone marrow–derived macrophage; IFNγ, interferon-γ; LPS, lipopolysaccharide.

Ribosome-associated translational stress has recently been implicated in the regulation of cytokine expression in macrophages following RNase L activation (17). As an alternative to direct detection of ribosome collisions, we employed two complementary strategies to functionally assess whether perturbations in ribosome dynamics can influence inflammatory responses. In the first approach, we treated resting macrophages with the translation elongation inhibitors cycloheximide (CHX) and ANS. Subinhibitory concentrations of translation elongation inhibitors have been shown to induce ribosome pausing and ribosome collisions by slowing elongation while permitting continued translation (18) (Fig. 4*A*). We therefore titrated CHX and ANS to identify doses that did not compromise macrophage viability or completely shut down translation (Fig. 4, *B* and *C*). Using these optimized conditions, we observed that translational stress induced by ANS was sufficient to induce expression of IL-6, TNF-α and IL-1β, while CHX only increased the expression of IL-6 (Fig. 4, *D*–*F*).

In the second approach, we disrupted the ribosome quality control (RQC) pathway, which normally functions to resolve stalled or collided ribosomes and limit the downstream consequences of aberrant translation (18, 19). Specifically, we used lentiviral transduction of short hairpin RNA (shRNA) to silence *Znf598* and reduce expression of the collision sensor ZNF598 (Fig. S4*A*), an E3 ubiquitin ligase that detects collided ribosomes and initiates RQC by ubiquitinating small ribosomal subunit proteins (14, 15, 16). When ZNF598-deficient macrophages were stimulated with LPS + IFNγ, they displayed a pronounced increase in the expression of the cytokines IL-6, TNF-α and IL-1β when compared with controls (Fig. 4, *G*–*I*). Consistent with these findings, quantitative PCR analysis revealed a corresponding increase in the mRNA levels of *Tnf* and *Il1b*, with a similar but non-significant trend for *Il6*, in ZNF598-deficient macrophages following LPS + IFNγ stimulation (Fig. S4, *B*–*D*). These findings indicate that the enhanced cytokine production is accompanied, at least in part, by increased transcriptional activity and is not solely attributable to translational regulation.

Our data indicate that perturbations in ribosome elongation dynamics, including conditions known to promote ribosome collisions such as CHX and ANS treatment, are sufficient to modulate inflammatory cytokine expression. In addition, silencing of ZNF598, whose primary known function is to sense collided ribosomes and activate the RQC pathway to resolve these events, led to increased inflammatory activation in M(LPS + IFNγ) macrophages. Together, these findings suggest that inflammatory macrophages accumulate ribosome collisions. Given that amino acid starvation does not appear to account for GCN2 activation in this context, we propose that GCN2 is instead activated downstream of ribosomal collisions.

### Loss of GCN2 accelerates and prolongs inflammatory programming

**Figure 5:**
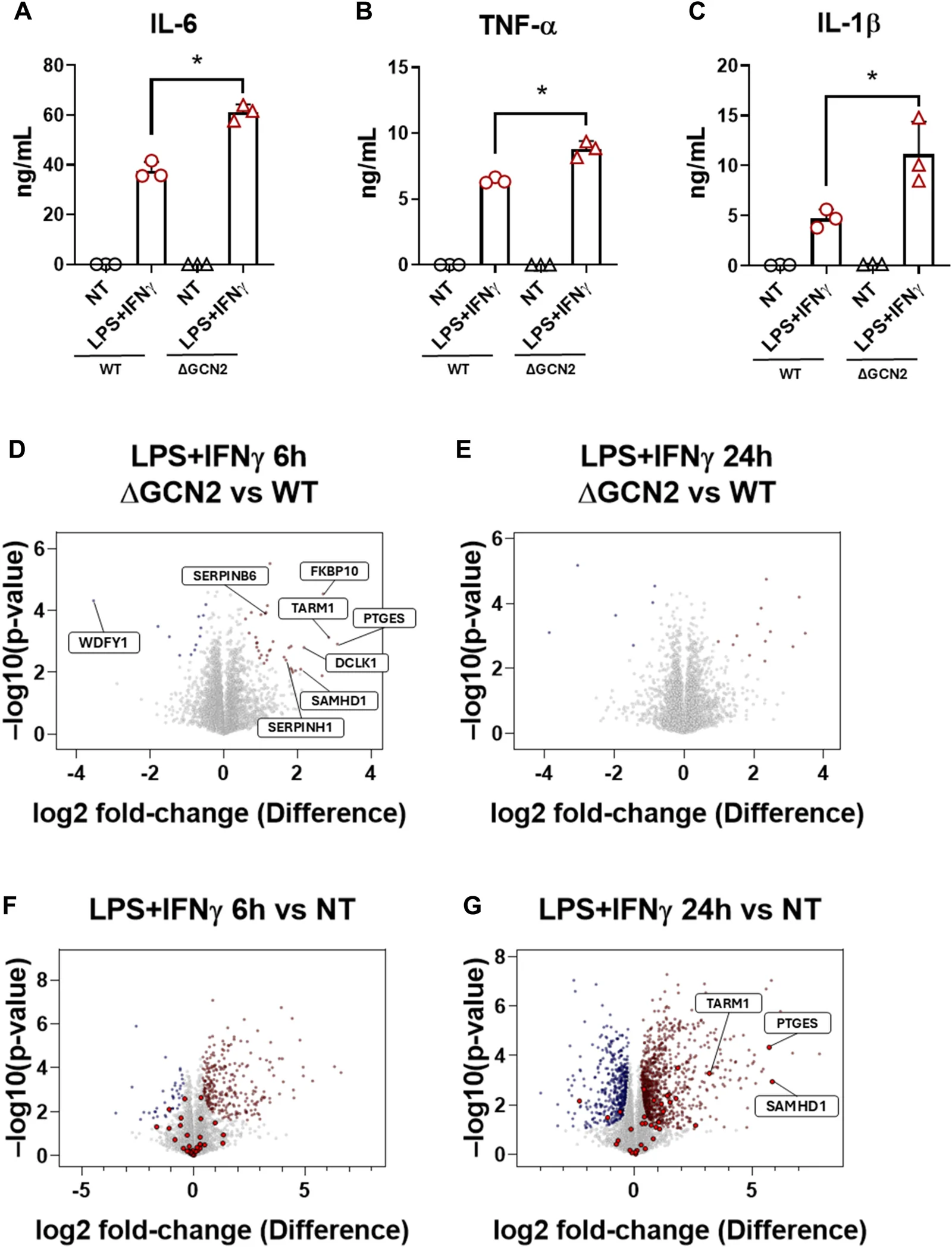
**Inflammatory profile of ΔGCN2 macrophages.***A–C,* cytokine expression measured by ELISA and quantitative PCR in WT and ΔGCN2 BMDMs after 24 h of LPS + IFNγ stimulation, highlighting the impact of GCN2 on inflammatory gene expression (n = 3). *D and E,* proteomic comparison of ΔGCN2 and WT BMDMs after 6h and 24h of LPS + IFNγ stimulation. *F and G,* proteomic comparison of WT BMDMs stimulated with LPS + IFNγ *versus* NT controls at 6 h and 24 h. Proteins highlighted in red are those upregulated in ΔGCN2 *versus* WT BMDMs at 6 h, which correspond to several proteins normally upregulated only at 24 h in WT cells, indicating earlier inflammatory activation in the absence of GCN2. Data are presented as mean ± SEM. Statistical significance was determined by two-way ANOVA with Tukey’s multiple comparisons test for *(A–C)*. ∗ = *p* < 0.05. BMDM, bone marrow–derived macrophage; GCN2, General Control Nonderepressible 2; IFNγ, interferon-γ; LPS, lipopolysaccharide; NT, non-treated.

Next, we determined how this translational repression shapes the inflammatory response. Cytokine measurements showed that ΔGCN2 M(LPS + IFNγ) macrophages secreted significantly higher levels of IL-6, IL-1β, and TNFα compared to controls after 24h (Fig. 5, *A*–*C*). This hyperinflammatory phenotype aligns with our data on ANS and ZNF598 silencing, as well as with previous reports in ΔGCN2 mice and BMDMs, which similarly showed increased transcription of proinflammatory cytokine genes accompanied by enhanced cytokine secretion (10).

Proteomic profiling of WT and ΔGCN2 M(LPS + IFNγ) macrophages at 6 and 24 h post-stimulation revealed that GCN2 deletion alters the inflammatory programming (Fig. 5, *D* and *E*). At the 6-h time point, ΔGCN2 M(LPS + IFNγ) macrophages exhibited a dysregulated proteome, including the premature upregulation of proinflammatory factors such as PTGES, SAMHD1, and TARM1 (Fig. 5*D*), which are not normally induced until 24 h of M(LPS + IFNγ) activation in WT cells (Fig. 5, *F* and *G*). By 24 h, however, ΔGCN2 macrophages did not exhibit a markedly stronger inflammatory profile compared to WT cells (Fig. 5*E*), indicating that loss of GCN2 primarily accelerates the onset rather than amplifies the overall magnitude of the inflammatory program. Consequently, ΔGCN2 macrophages experience a prolonged period of inflammatory activation, which results in increased proinflammatory output over time.

### ZAKα selectively mediates TNFα hyperproduction in ΔGCN2 macrophages

**Figure 6:**
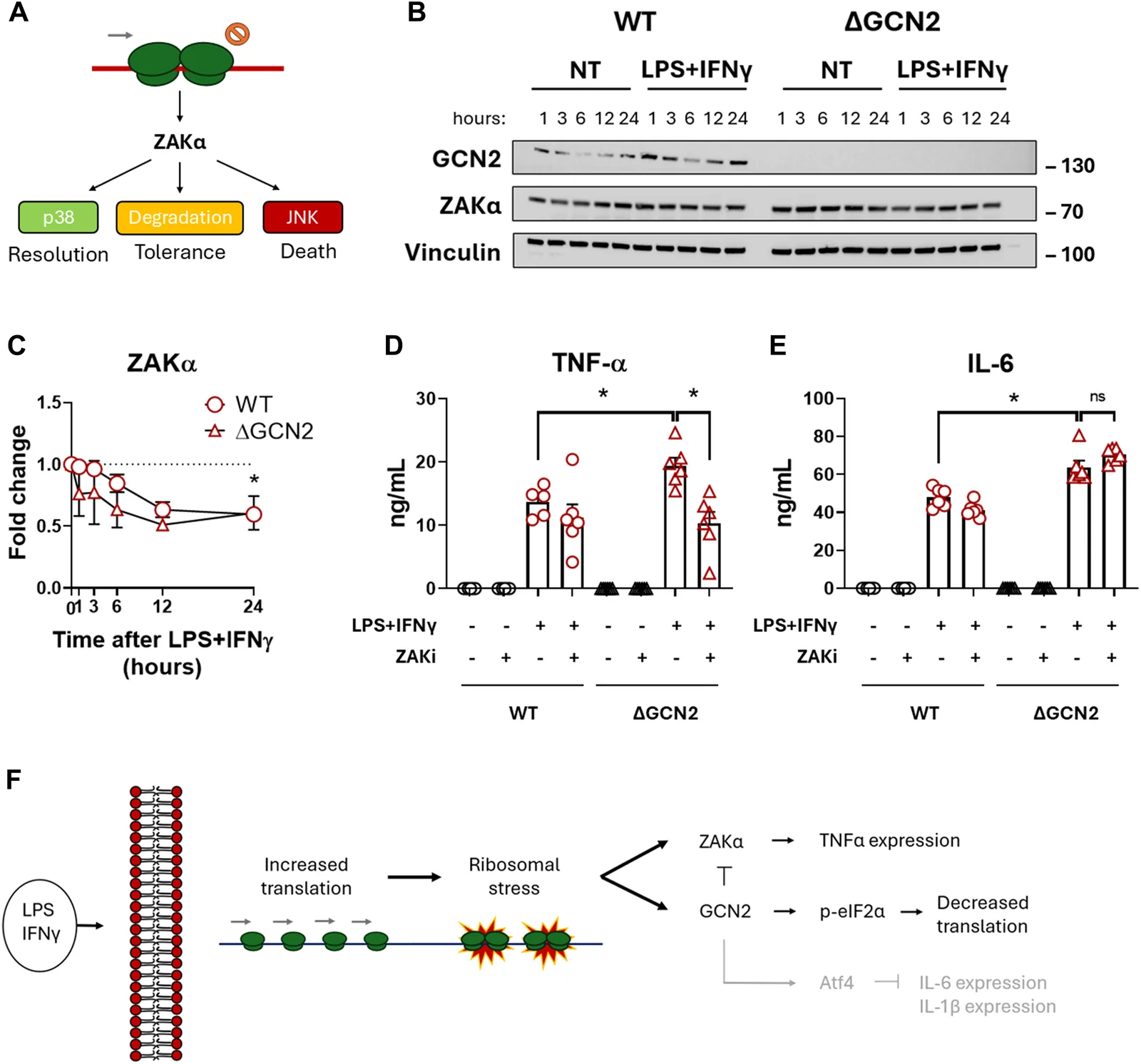
**Role of ZAKα in inflammatory responses of ΔGCN2 macrophages.***A,* schematic of ZAKα activation by ribosome collisions and possible outcomes. *B and C,* immunoblot analysis of ZAKα expression in WT and ΔGCN2 BMDMs treated with LPS + IFNγ over time, showing decreased expression of ZAKα after 24 h (n = 3). *D and E,* cytokine expression measured by ELISA in WT and ΔGCN2 BMDMs after 24 h of LPS + IFNγ stimulation, with or without ZAKα inhibition, showing that ZAKα inhibition reverses the effect of GCN2 deficiency on TNFα production, while IL-6 levels remain unaltered (n = 6). *F,* proposed model: LPS + IFNγ stimulation induces translational stress, which in turn activates GCN2 to restrain protein synthesis through eIF2α phosphorylation. In the absence of GCN2, ZAKα becomes overactivated, leading to dysregulated signaling and elevated TNFα expression. Data are presented as mean ± SEM *(n = 3 for B, C; n = 6 for D, E)*. Statistical significance was determined by two-way ANOVA with Tukey’s multiple comparisons test for *(B–E)*. ∗ = *p* < 0.05. BMDM, bone marrow–derived macrophage; eIF2α, eukaryotic translation initiation factor 2α; GCN2, General Control Nonderepressible 2; IFNγ, interferon-γ; LPS, lipopolysaccharide.

Given that translational elongation stress associated with perturbations in ribosome dynamics can engage multiple ribosome-associated stress response pathways, including GCN2 and the ribotoxic stress sensor ZAKα (8), we investigated whether a functional relationship exists between these two kinases during macrophage activation. ZAKα has been shown to respond to ribosome-associated stress, with the abundance and persistence of such stress determining distinct cellular outcomes, including stress resolution, tolerance, or cell death (8, 9, 20, 21, 22) (Fig. 6*A*).

Because macrophages remain viable following prolonged inflammatory stimulation, we reasoned that they preferentially engage a ZAKα-associated tolerance program, which has been linked to ZAKα downregulation after ribotoxic stress (9). Consistent with this model, ZAKα protein levels decreased by approximately 20% after 24 h of M(LPS + IFNγ) activation (Fig. 6, *B* and *C*). ZAKα levels showed a modest trend toward further reduction in ΔGCN2 macrophages, although this difference did not reach statistical significance. While not conclusive, this pattern is consistent with increased ribosome-associated stress in the absence of GCN2-mediated translational control, without compromising cell viability (data not shown). In line with engagement of a ZAKα tolerance-associated state (9), phosphorylation of the downstream MAPKs p38 and JNK was reduced in ΔGCN2 macrophages, indicating attenuated MAPK signaling (Fig. S5, *A*–*C*).

We attempted to assess ZAKα activation by monitoring phosphorylation of ZAKα; however, phosphorylated ZAKα was not detectable in primary macrophages under our experimental conditions, even in response to canonical ribotoxic stress controls (8, 9) (Fig. S5, *D* and *E*). We therefore relied on functional approaches to evaluate the contribution of ZAKα signaling.

Prior work attributed hyperinflammatory responses in ΔGCN2 macrophages to altered ATF4 activity, which regulates IL-6 and IL-1β but not TNFα (7). This distinction prompted us to investigate whether the exaggerated TNFα production observed in our ΔGCN2 macrophages was regulated through a ZAKα-dependent mechanism. Pharmacological inhibition of ZAKα with nilotinib completely normalized TNFα secretion in ΔGCN2 macrophages (Fig. 6*D*). Notably, nilotinib treatment also restored ZAKα protein levels in ΔGCN2 macrophages (Fig. S5, *F* and *G*), consistent with activity-dependent turnover of ZAKα and supporting the interpretation that reduced ZAKα abundance reflects engagement of a ZAKα-associated stress response rather than nonspecific protein loss. In contrast, ZAKα inhibition had no effect on IL-6 expression (Fig. 6*E*), consistent with the established ATF4-dependent regulation of this cytokine (7).These findings reveal a functionally distinct regulatory axis in which GCN2 constrains inflammatory output by limiting ZAKα-dependent TNFα production, while other cytokines such as IL-6 are regulated through parallel, ATF4-dependent pathways.

Together, our findings support a model in which LPS + IFNγ stimulation drives a rapid increase in protein synthesis, generating translational stress that engages multiple ribosome-associated stress response pathways, including GCN2 and ZAKα (Fig. 6*F*). Activation of GCN2 leads to phosphorylation of eIF2α, dampening global translation and thereby limiting the downstream consequences of ribosome-associated stress. In the absence of GCN2, failure to restrain translational load results in exaggerated ZAKα-dependent inflammatory signaling, particularly affecting TNFα production. This functional interplay between GCN2-mediated translational control and ZAKα-dependent inflammatory signaling thus represents a critical checkpoint for maintaining translational and inflammatory homeostasis in activated macrophages.

## Discussion

Macrophages must rapidly remodel their proteome to execute inflammatory responses, yet the overarching translational programs governing this process have remained elusive. In this study, we show that inflammatory and reparative macrophage activation is defined by fundamentally distinct translational dynamics. Moreover, we identify a critical regulatory axis centered on the stress-sensing kinase GCN2, which acts as a brake to prevent translational overload and exacerbated inflammation by restraining ZAKα-dependent inflammatory signaling.

We first demonstrate that proinflammatory and reparative activation drive divergent translational trajectories. While M(IL-4) macrophages sustain elevated levels of protein synthesis for days, M(LPS + IFNγ) macrophages execute a rapid, transient burst of translation that is followed by a sharp decline. This decline coincides with hallmarks of translational stress, including enrichment of P-body–associated proteins, suggesting engagement of a self-limiting translational program. We determine that this biphasic response is primarily gated by the eIF2α pathway. In contrast, M(IL-4) BMDMs show no clear relationship between translation rates and the phosphorylation status of either eIF2α or 4EBP, indicating that alternative mechanisms govern translational control in these cells. Potential candidates include eIF4E phosphorylation by MAPK-interacting kinases or cap-independent translation pathways. Indeed, eIF4E phosphorylation by MAPK-interacting kinase2 has previously been implicated in the regulation of the M(IL-4) BMDM phenotype (23).

Our data indicate that GCN2 is a major eIF2α kinase responsible for applying an early translational brake in inflammatory macrophages. Loss of GCN2 results in increased early translation and a hyperinflammatory phenotype characterized by premature and exaggerated cytokine production, including TNFα. This finding aligns with previous studies linking GCN2 deficiency to enhanced inflammation (7, 8), but the mechanistic basis for this effect, particularly with respect to TNFα, has remained unclear. We hypothesized that hyperinflammation in ΔGCN2 macrophages stems from unrestrained ribosome-associated translational stress. Consistent with this idea, independent perturbation of ribosome elongation, either pharmacologically or by disruption of RQC through ZNF598 depletion, was sufficient to enhance inflammatory cytokine expression, supporting the notion that ribosomal dysfunction itself acts as a proinflammatory signal.

Proteomic profiling revealed that ΔGCN2 macrophages exhibit altered inflammatory programming at both early and late stages of activation. At 6 h, ΔGCN2 macrophages displayed differential regulation of genes involved in macrophage activation across diverse contexts, including Wdfy1, Dclk1, Fkbp10, and members of the Serpin family (24, 25, 26, 27, 28, 29, 30, 31, 32), as well as premature upregulation of proinflammatory mediators such as Ptges (33, 34, 35), which are normally induced only at later stages in WT macrophages. These findings indicate that loss of GCN2 accelerates the inflammatory program rather than simply amplifying its magnitude.

We further identify ZAKα as a key mediator linking ribosome-associated stress to TNFα-specific hyperproduction. In WT macrophages, the reduction of ZAKα protein levels and attenuation of downstream MAPK signaling are consistent with engagement of a ZAKα-associated tolerance program. In ΔGCN2 macrophages, this pathway appears dysregulated, resulting in sustained ZAKα-dependent inflammatory signaling. Importantly, pharmacological inhibition of ZAKα with nilotinib specifically normalized TNFα production in ΔGCN2 macrophages without affecting IL-6 expression, thereby resolving a key ambiguity in the field. Previous work established that GCN2-mediated ATF4 activation regulates IL-6 and IL-1β (7); our data demonstrate that TNFα is uniquely constrained through a ZAKα-dependent pathway. Thus, GCN2 may suppress inflammation through coordinated regulation of both the ATF4 arm of the integrated stress response and the ZAKα arm of the ribotoxic stress response.

While ZAKα has been implicated in cytokine production under conditions of extreme ribotoxic stress induced by toxins such as ricin (36, 37), its role in the context of physiological inflammatory stimulation was previously unclear. Our findings demonstrate that canonical inflammatory signaling downstream of Toll-like receptor four and IFNγ receptor engagement is sufficient to generate ribosome-associated stress, and that the GCN2–ZAKα axis functions as an important checkpoint to maintain inflammatory homeostasis. The effect of nilotinib in resolving TNFα-driven hyperinflammation highlights the therapeutic potential of this pathway. Notably, nilotinib has been previously described as an inhibitor of inflammatory activity, though the underlying mechanisms remained poorly defined. In RAW macrophages, nilotinib was shown to reduce iNOS expression and increase IL-6 production through mechanisms involving p38 and JNK activation, without affecting NF-κB signaling (38). In microglia, nilotinib modulated IL-1β and IL-6 expression in a p38-and SOD2-dependent manner, suggesting a role for oxidative stress in this regulation (39). Given the central roles of p38 and JNK in inflammatory signaling (40, 41, 42), our findings position ZAKα as a key upstream regulator linking ribosome-associated stress to cytokine output.

High levels of reactive oxygen species (ROS) have been reported to induce ribosome collisions, leading to inhibition of global protein synthesis and activation of RQC pathways and ZAKα in other cellular systems (43, 44). Increased ROS production is a hallmark of macrophage activation downstream of TLR signaling, where it contributes to bactericidal activity (45, 46). Similarly, nitric oxide (NO), another hallmark of proinflammatory macrophage activation (47, 48), has been shown to induce ribosome collisions (49). It is therefore plausible that the translational stress signatures and repression of protein synthesis observed in our system are driven, at least in part, by ROS and NO production following inflammatory stimulation. Future studies will be required to directly test this possibility.

In conclusion, we propose a model in which proinflammatory macrophage activation inherently generates ribosome-associated translational stress. GCN2 senses this stress and phosphorylates eIF2α to globally restrain translation initiation, thereby mitigating proteotoxic load and limiting excessive inflammatory signaling. In the absence of GCN2, failure to restrain translational demand results in exaggerated ZAKα-dependent TNFα production. This feedback mechanism positions translational control as a central checkpoint governing the intensity and duration of macrophage inflammatory responses. Targeting the GCN2–ZAKα axis may therefore represent a promising strategy for treating inflammatory diseases characterized by dysregulated TNFα production.

### Limitations of the study

While our findings provide important insights into how translational control shapes macrophage inflammatory responses, several limitations should be acknowledged. First, this study was primarily conducted in murine macrophages, and future work will be required to determine the extent to which these mechanisms operate in human macrophages and *in vivo* inflammatory settings.

Second, although our data support a role for ribosome-associated translational stress in driving inflammatory signaling, we did not directly visualize or biochemically quantify ribosome collisions in activated macrophages. Direct detection of ribosome collisions remains technically challenging in primary immune cells under physiological stimulation conditions. Therefore, our conclusions rely on convergent functional evidence, including perturbation of translational elongation, disruption of RQC, and pharmacological inhibition of ZAKα.

Third, while we speculate that ROS or NO produced during inflammatory activation may contribute to translational stress, we did not directly test this possibility in our system.

Finally, although we identify ZAKα as a key mediator of TNFα regulation downstream of translational stress, additional stress-responsive kinases and pathways may also participate in coordinating translational and inflammatory responses in macrophages and their contributions warrant further investigation.

## Experimental procedures

### Mice

WT C57BL/6J mice at UNICAMP were maintained in collective microisolators with wood shavings, housing up to five animals of the same sex per cage, under a 12/12-h light/dark cycle, constant temperature (22 °C), and ad libitum access to filtered water and autoclaved standard chow. ΔGCN2 (B6.129S6-Eif2ak4tm1.2Dron/J) and WT mice at the Max Planck Institute of Biochemistry were housed in open cages within a large room accommodating 200 to 250 cages, under the same conditions of light cycle, temperature (22 °C), and food and water availability. The use of mice for bone marrow isolation and generation of BMDMs at UNICAMP was approved by the Animal Ethics Committee of the Institute of Biology, University of Campinas (CEUA; protocol no. 5856-1/2021). The use of mice for bone marrow isolation and generation of BMDMs at the Max Planck Institute of Biochemistry was approved by the Regierung von Oberbayern animal ethics committee.

### Bone marrow-derived macrophages culture

BMDMs were obtained from ΔGCN2 (B6.129S6-Eif2ak4^tm1.2Dron^/J; The Jackson Laboratory) or WT C57BL/6 mice by flushing bone marrow cells from femurs and tibias under sterile conditions, as described. Isolated cells were cultured and differentiated for 7 days in Dulbecco’s Modified Eagle Medium (DMEM, high glucose) supplemented with 10% heat-inactivated fetal bovine serum, 1% penicillin–streptomycin, and either 20 ng/ml recombinant macrophage colony-stimulating factor or 10% L929 cell-conditioned medium, following the protocol described (50). Differentiation medium was replaced every 2 to 3 days, and cultures were maintained at 37 °C in a humidified incubator with 5% CO_2_ until macrophages were fully matured. For polarization and activation experiments, BMDMs were stimulated with lipopolysaccharide (LPS, 100 ng/ml) in combination with interferon-γ (IFNγ, 20 ng/ml) to induce classically activated macrophages, or with interleukin-4 (IL-4, 20 ng/ml) to induce alternatively activated macrophages. Cells were treated for varying durations depending on the experimental design. To evaluate stress responses, translational stress was induced by treatment with CHX (Sigma) or ANS, Sigma at concentrations ranging from 0.1 to 10 μg/ml. For inhibition of ZAKα kinase activity, cells were incubated with nilotinib (APExBIO, 1 μg/ml).

### Western blot

Cells were lysed on ice using radioimmunoprecipitation assay buffer (50 mM Tris-HCl, pH 7.4; 150 mM NaCl; 1% NP-40; 0.5% sodium deoxycholate (SDC); 0.1% SDS) freshly supplemented with a protease inhibitor cocktail and phosphatase inhibitors (Thermo Fisher Scientific). Lysates were incubated on ice for 30 min with intermittent vortexing and subsequently cleared by centrifugation at 12,000 × *g* for 15 min at 4 °C to remove cell debris. Protein concentrations were quantified using the bicinchoninic acid protein assay kit (Thermo Fisher Scientific) according to the manufacturer’s instructions. Equal amounts of protein (20–30 μg per sample) were denatured in Laemmli sample buffer, resolved on SDS–polyacrylamide gels, and transferred onto polyvinylidene difluoride membranes (Millipore) using a wet transfer system. Following transfer, membranes were blocked in 3% milk or 1% bovine serum albumin in Tris-buffered saline containing 0.1% Tween-20 for 1 h at room temperature to reduce nonspecific binding. Membranes were then incubated overnight at 4 °C with the indicated primary antibodies, diluted in blocking buffer at concentrations recommended by the supplier. After three washes with Tween-20, membranes were incubated with horseradish peroxidase-conjugated secondary antibodies for 1 h at room temperature. Immunoreactive bands were visualized using a chemiluminescence detection system (Thermo Fisher Scientific) and imaged with a ChemiDoc imaging system (Bio-Rad). Densitometric quantification of protein bands was performed using ImageJ software (NIH; https://imagej.net/), and protein expression levels were normalized to the appropriate loading controls. For the puromycin incorporation assay, cells were incubated with puromycin (10 μg/ml) in pre-warmed culture medium for 20 min at 37 °C prior to protein extraction. Following incubation, cells were washed twice with cold phosphate-buffered saline and lysed as described above. Puromycin incorporation into nascent polypeptides was detected by Western blot using an anti-puromycin antibody, and quantification was performed by densitometric analysis as described above.

### Phos-tag gel electrophoresis

Cells were lysed and protein samples were prepared as described above. For detection of ZAKα phosphorylation, proteins were resolved on 8% SDS–polyacrylamide gels supplemented with Phos-tag acrylamide (30 μM) and ZnCl_2_ (100 μM). Electrophoresis was performed at 90 V for 2 h using a Tris/MOPS/SDS running buffer (pH 7.8; 5 × stock: 0.5 M Tris, 0.5 M MOPS, 0.5% SDS; diluted 1 × with 0.5 M NaHSO_3_ immediately before use). Following separation, gels were washed twice for 10 min in transfer buffer (25 mM Tris, 192 mM glycine, 10% methanol) containing 1 mM EDTA, followed by two washes in transfer buffer without EDTA. Proteins were transferred to polyvinylidene difluoride membranes (Bio-Rad) using a wet transfer system at 100 V for 1 h 30 min at room temperature.

### Quantitative PCR

Total RNA was extracted from cells using TRIzol reagent (Invitrogen) according to the manufacturer’s protocol. After phase separation, RNA was precipitated with isopropanol, washed with 75% ethanol, and resuspended in RNase-free water. RNA concentration and purity were measured using a NanoDrop spectrophotometer (Thermo Fisher Scientific), and samples with A260/A280 ratios between 1.8 and 2.0 were used for subsequent experiments. RNA integrity was further verified by agarose gel electrophoresis. Complementary DNA was synthesized from 500 to 1000 ng of total RNA using oligo(dT) primers, random hexamers, and MultiScribe Reverse Transcriptase (Applied Biosystems), according to the manufacturer’s instructions. Quantitative PCR was performed using SYBR Green Master Mix (Applied Biosystems) on a real-time PCR detection system (*e.g.*, StepOnePlus, Applied Biosystems). The cycling program consisted of an initial denaturation at 95 °C for 10 min, followed by 40 cycles of 95 °C for 15 s and 60 °C for 1 min. A dissociation curve was included to confirm primer specificity and absence of primer-dimer formation. Relative gene expression was calculated using the 2^ˆ^−ΔΔCt method, with 18S rRNA serving as the endogenous control. All reactions were carried out in technical triplicates, and data were obtained from at least three independent biological replicates.

### ELISA

Culture supernatants were collected at the indicated time points, centrifuged at 500 × *g* for 5 min to remove cell debris, and stored at −80 °C until further analysis. Concentrations of the cytokines were quantified using commercially available ELISA kits (BioLegend) according to the manufacturer’s protocols. Briefly, 96-well plates pre-coated with capture antibodies specific for each cytokine were incubated overnight at 4 °C. Plates were then washed and blocked with assay buffer for 1 h at room temperature to minimize nonspecific binding. Standards and appropriately diluted culture supernatants were added in duplicate and incubated for 2 h at room temperature. After washing, biotinylated detection antibodies were added, followed by incubation with streptavidin– horseradish peroxidase. The enzymatic reaction was developed with TMB substrate, stopped with 2N H_2_SO_4_, and absorbance was measured at 450 nm using a microplate reader (BioTek). Cytokine concentrations were determined from standard curves generated with recombinant cytokines provided in the kits.

### Lentiviral transduction

A second-generation lentiviral plasmid system was used for viral transfection, consisting of the packaging plasmid psPAX2, the envelope plasmid pMD2.G, and the shRNA expression plasmid pLKO.1. pLKO.1 vectors carrying shRNA sequences targeting the ZNF598 gene were prepared, as well as targeting GFP as control. HEK293T cells were seeded at 3,5 × 10^6^ cells per well in 10 cm plates and incubated for 18 to 24 h. Cells were then transfected with a mixture of PEI and the three plasmids, followed by an additional 6 to 8 h of incubation. The medium was replaced with 10 ml of fresh medium, and viral supernatants were collected at 48 h post-transfection. Supernatants were centrifuged at 500 × *g* for 5 min, filtered through a 0.45 μm PES filter, snap-frozen in liquid nitrogen, and stored at −80 °C. For transduction, BMDMs were infected with lentivirus on day 1 of culture and centrifuged for 1h30 at 32 °C in the presence of polybrene (8 μg/ml), followed by 48 h incubation. Medium was then replaced with fresh medium containing 1 μg/ml puromycin for selection, with medium changes as needed. Gene silencing efficiency was assessed by quantitative PCR. Macrophages were subsequently stimulated with LPS + IFNγ for 24 h, or left untreated as controls.

### Flow cytometry

Cell viability and apoptosis were assessed using the Pacific Blue Annexin V Apoptosis Detection Kit with 7-aminoactinomycin D(BioLegend) following the manufacturer’s instructions. Briefly, adherent and/or suspension cells were collected, washed twice with ice-cold phosphate-buffered saline, and resuspended in Annexin V binding buffer at a concentration of 1 × 10^6^ cells/ml. Cells were then incubated with Pacific Blue–conjugated Annexin V and 7-aminoactinomycin D for 15 min at room temperature in the dark to prevent photobleaching. After staining, cells were immediately analyzed using a flow cytometer (*e.g.*, BD FACS Symphony A5) data were processed and analyzed using FlowJo software (v10, BD; https://www.flowjo.com/), and results are presented as the percentage of cells in each population.

### LC–MS/MS analysis

Proteins were denatured by adding 80 μl of SDC buffer containing 1% SDC; Sigma-Aldrich, 40 mM 2-chloroacetamide (Sigma-Aldrich), 10 mM tris(2-carboxyethyl) phosphine (Thermo Fisher Scientific), and 100 mM Tris (pH 8.0). The mixture was incubated for 30 min at 37 °C. The samples were diluted 1:1 with water. Proteins were then digested overnight at 37 °C with 0.5 μg trypsin (Promega). Following digestion, peptides were acidified with TFA; Merck to a final concentration of 1%. Peptides were either desalted using SCX StageTips or directly loaded (200 ng total peptide) onto Evotips (Evotip Pure; Evosep). Peptides were eluted from Evotips onto a 15 cm PepSep C18 column (15 cm × 150 μm, 1.5 μm; Bruker Daltonics) using an Evosep One HPLC system. The column was maintained at 50 °C, and peptides were separated using the 30 SPD method. Data were acquired on a timsTOF Pro mass spectrometer operated with timsControl software in data-independent acquisition (DIA) PASEF mode. The MS scan range was 100 to 1700 m/z with an ion mobility range of 1/K_0_ = 0.70 to 1.30 V cm^−2^. Equal ion accumulation and ramp times of 100 ms were used in the dual TIMS analyzer, yielding a spectral rate of 9.52 Hz. DIA-PASEF scans were acquired in the 350.2 to 1199.9 Da range with 42 DIA-PASEF windows assigned to one TIMS scan each. Precursor isolation windows were alternated, resulting in an overall cycle time of 2.21 s. The collision energy was linearly ramped from 45 eV at 1/K_0_ = 1.30 V cm^−2^ to 27 eV at 1/K_0_ = 0.85 V cm^−2^. Raw data were processed in Spectronaut 18.0 (Biognosys) using directDIA+ (library-free) mode. Spectra were searched against a predicted *Mus musculus* protein database from UniProt (SwissProt and TrEMBL; downloaded 2023). Cysteine carbamidomethylation was set as a fixed modification, and methionine oxidation and protein N-terminal acetylation were set as variable modifications. Protein quantification across samples was performed using label-free quantification (MaxLFQ) at the MS (2) level.

## Data availability

The mass spectrometry proteomics data have been deposited to the ProteomeXchange Consortium *via* the PRIDE partner repository with the dataset identifier PXD075112.

## Supporting information

This article contains supporting information.

## Conflict of interest

The authors declare that they have no conflicts of interest with the contents of this article.
