# Supplementary material for: The integrated stress response kinase GCN2 prevents ZAKα-Dependent inflammatory hyperactivation in macrophages
Source: J Biol Chem. 2026 Aug 14;302(10):113452. doi: 10.1016/j.jbc.2026.113452 (PMC13584029; doi:10.1016/j.jbc.2026.113452)
Supplement: Supplementary file 1 [file mmc1.pdf]

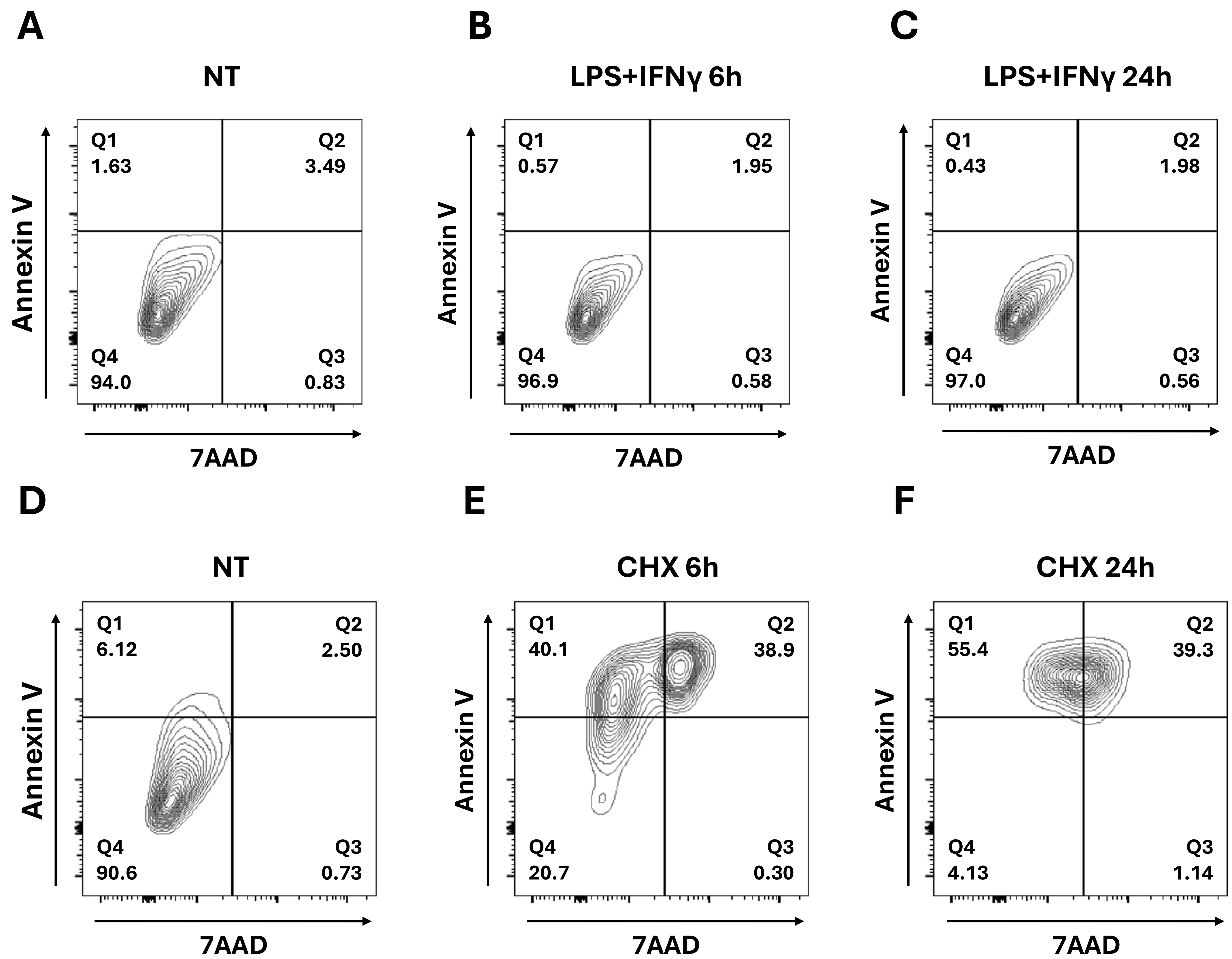
A
NT
Q1 1.63
Q2 3.49
Q4 94.0
Q3 0.83
Annexin V
7AAD
B
LPS+IFNγ 6h
Q1 0.57
Q2 1.95
Q4 96.9
Q3 0.58
Annexin V
7AAD
C
LPS+IFNγ 24h
Q1 0.43
Q2 1.98
Q4 97.0
Q3 0.56
Annexin V
7AAD
D
NT
Q1 6.12
Q2 2.50
Q4 90.6
Q3 0.73
Annexin V
7AAD
E
CHX 6h
Q1 40.1
Q2 38.9
Q4 20.7
Q3 0.30
Annexin V
7AAD
F
CHX 24h
Q1 55.4
Q2 39.3
Q4 4.13
Q3 1.14
Annexin V
7AAD

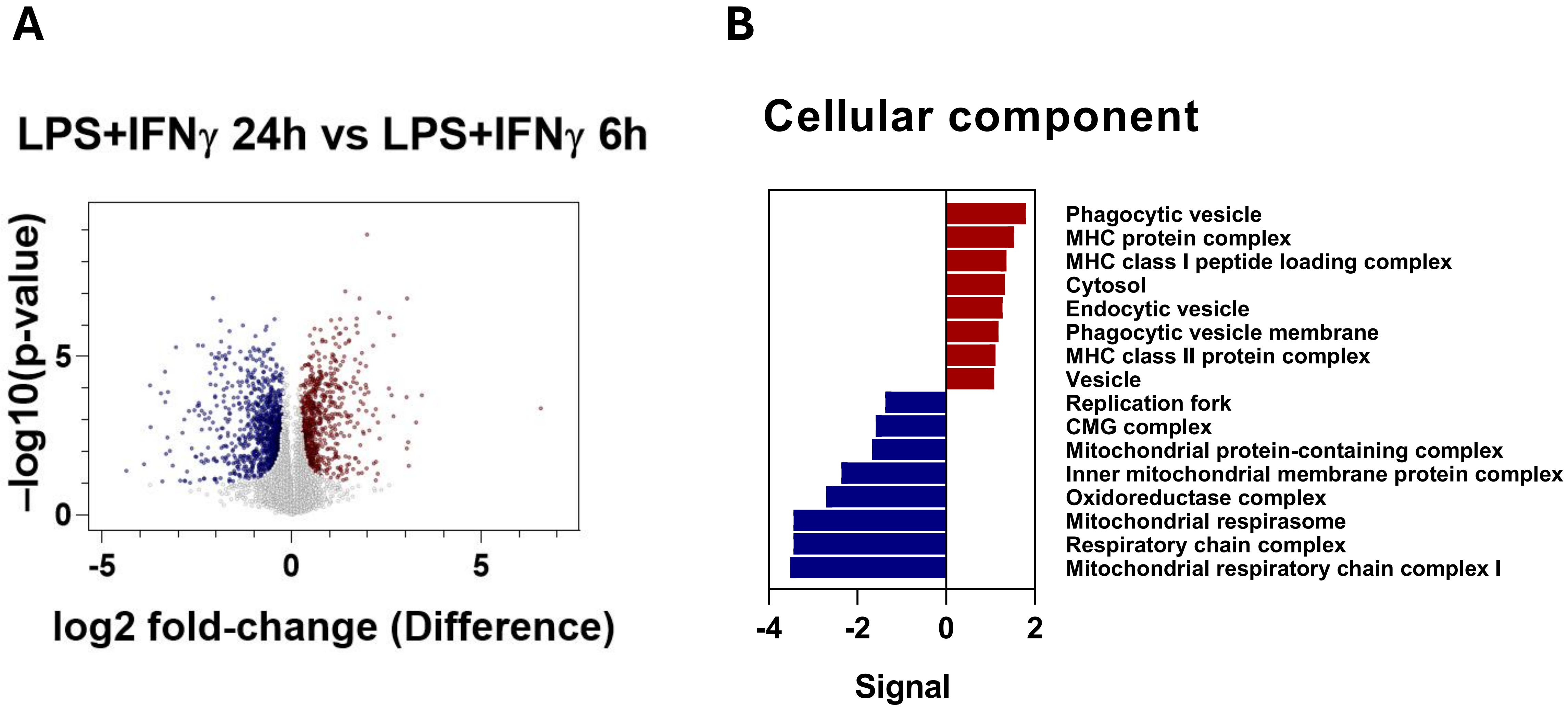
A
LPS+IFNγ 24h vs LPS+IFNγ 6h
−log10(p-value)
5
0
-5
0
5
log2 fold-change (Difference)
B
Cellular component
Phagocytic vesicle
MHC protein complex
MHC class I peptide loading complex
Cytosol
Endocytic vesicle
Phagocytic vesicle membrane
MHC class II protein complex
Vesicle
Replication fork
CMG complex
Mitochondrial protein-containing complex
Inner mitochondrial membrane protein complex
Oxidoreductase complex
Mitochondrial respirasome
Respiratory chain complex
Mitochondrial respiratory chain complex I
-4
-2
0
2
Signal

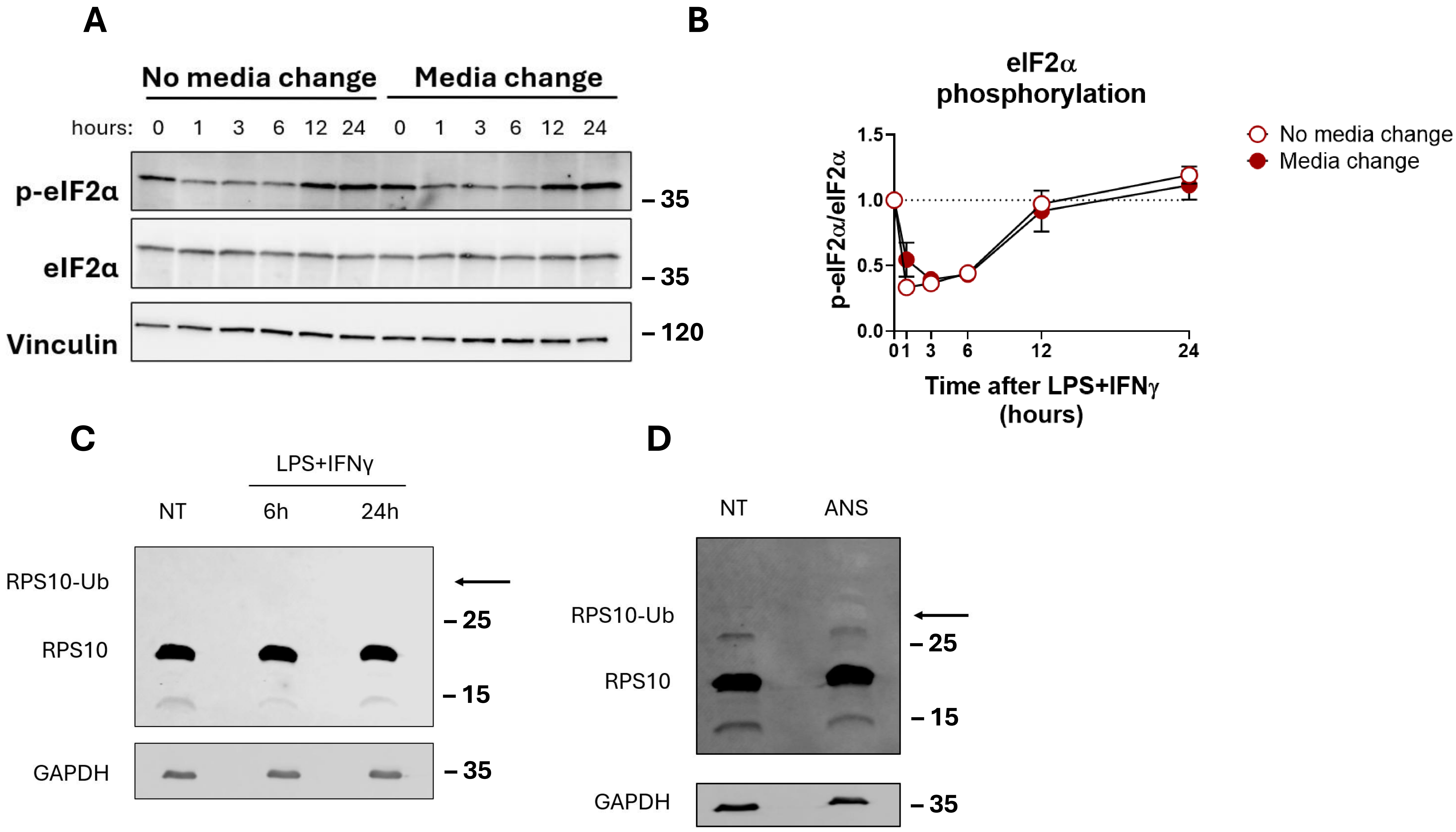
A
No media change
Media change
hours: 0 1 3 6 12 24 0 1 3 6 12 24
p-eIF2α
eIF2α
Vinculin
35
35
120
B
eIF2α phosphorylation
p-eIF2α/eIF2α
1.5
1.0
0.5
0.0
0 1 3 6 12 24
Time after LPS+IFNγ (hours)
No media change
Media change
C
LPS+IFNγ
NT 6h 24h
RPS10-Ub
RPS10
25
15
GAPDH
35
D
NT ANS
RPS10-Ub
RPS10
25
15
GAPDH
35

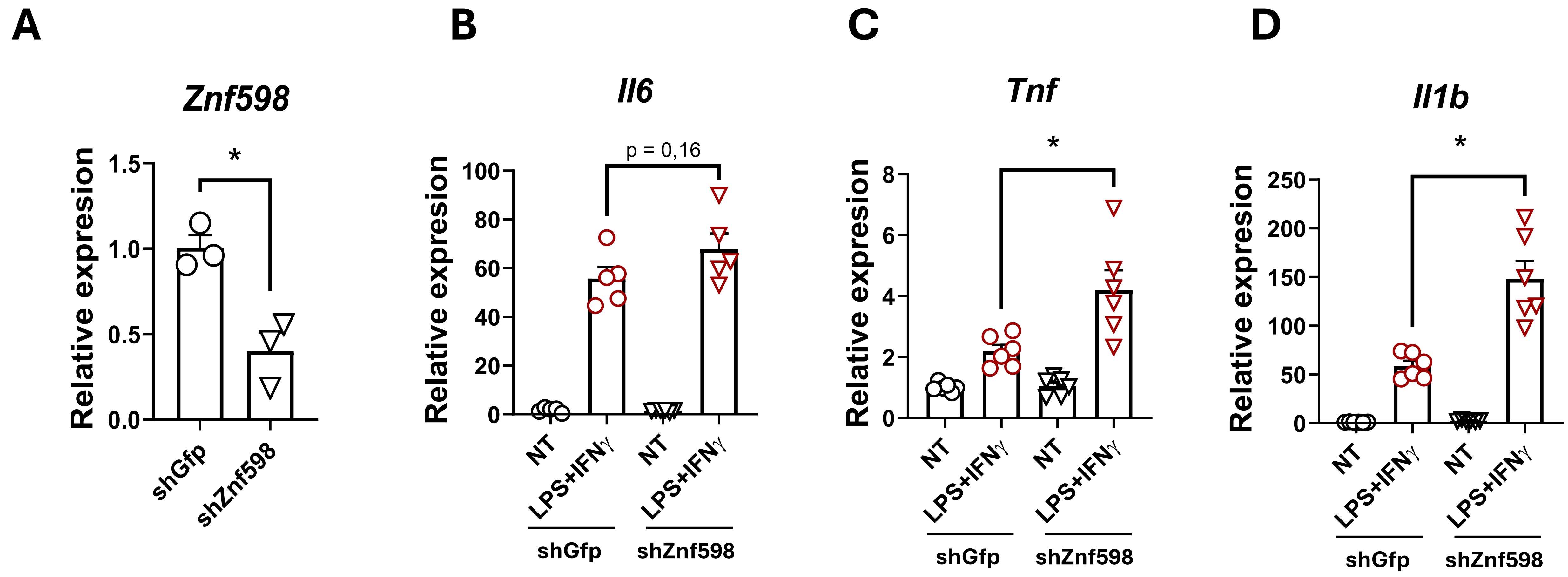

A
Znf598
*
Relative expresion
1.5
1.0
0.5
0.0
shGfp
shZnf598
B
Il6
p = 0,16
Relative expresion
100
80
60
40
20
0
NT
LPS+IFNγ
NT
LPS+IFNγ
shGfp
shZnf598
C
Tnf
*
Relative expresion
8
6
4
2
0
NT
LPS+IFNγ
NT
LPS+IFNγ
shGfp
shZnf598
D
Il1b
*
Relative expresion
250
200
150
100
50
0
NT
LPS+IFNγ
NT
LPS+IFNγ
shGfp
shZnf598

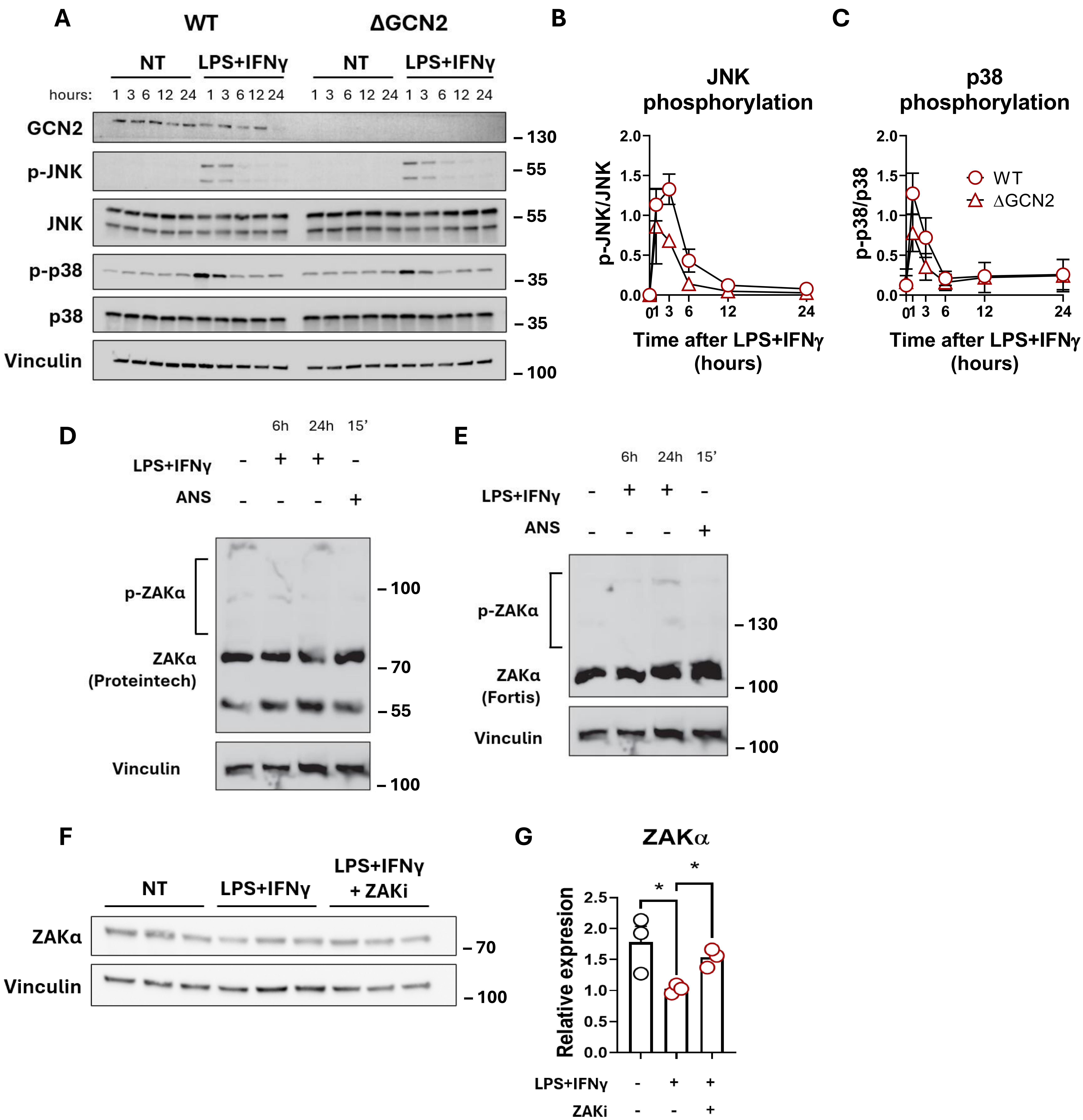
A
WT
ΔGCN2
NT
LPS+IFNγ
NT
LPS+IFNγ
hours: 1 3 6 12 24 1 3 6 12 24 1 3 6 12 24 1 3 6 12 24
GCN2
p-JNK
JNK
p-p38
p38
Vinculin
– 130
– 55
– 55
– 35
– 35
– 100
B
JNK phosphorylation
p-JNK/JNK
Time after LPS+IFNγ (hours)
C
p38 phosphorylation
p-p38/p38
WT
ΔGCN2
Time after LPS+IFNγ (hours)
D
6h 24h 15'
LPS+IFNγ - + + -
ANS - - - +
p-ZAKα
ZAKα (Proteintech)
Vinculin
– 100
– 70
– 55
– 100
E
6h 24h 15'
LPS+IFNγ - + + -
ANS - - - +
p-ZAKα
ZAKα (Fortis)
Vinculin
– 130
– 100
– 100
F
NT
LPS+IFNγ
LPS+IFNγ + ZAKi
ZAKα
Vinculin
– 70
– 100
G
ZAKα
Relative expresion
LPS+IFNγ - + +
ZAKi - - +

Figure S1. Cell viability in BMDMs following pro-inflammatory stimulation. (A-C) Flow cytometry analysis of BMDM viability after stimulation with LPS+IFNγ for 6h and 24h, assessed by AnnexinV and 7AAD staining. (D-F) Cycloheximide treatment (10μg/mL) was used as a positive control for cell death.

Figure S2. Temporal proteome analysis of M(LPS+IFNγ) macrophages. (A) Proteomic analysis of BMDMs stimulated with LPS+IFNγ for 24h compared with BMDMs stimulated for 6h. (B) Gene ontology (GO) pathway analysis of differentially expressed proteins identified in (A).

Figure S3. Additional analyses of eIF2α regulation and ribosome quality control during inflammatory activation. (A, B) Immunoblot analysis of eIF2α phosphorylation over time following LPS+IFNγ stimulation with or without media change. Quantification is shown in (B) (n = 2), showing no detectable differences between conditions. (C) RSP10 expression following LPS+IFNγ stimulation; black arrow represents expected size of RSP10-ub. (D) Immunoblot of BMDMs stimulated with LPS+IFNγ showing total RPS10. The black arrow marks the expected position of ubiquitinated RPS10 (RPS10-Ub). (E) Immunoblot analysis of RPS10 after ANS treatment (2 μM, 15 min).; the expected molecular weight of ubiquitinated RPS10 (RPS10-Ub) is indicated, but no detectable ubiquitinated species was observed under these conditions.

Figure S4. Validation of ZNF598 knockdown and transcriptional analysis of inflammatory cytokines. (A) Confirmation of *Znf598* knockdown efficiency by qPCR. (B–D) Relative mRNA expression of *Il6* (B), *Tnf* (C), and *Il1b* (D) in control and ZNF598-deficient macrophages following LPS+IFNγ stimulation. Data are presented as mean ± SEM (n = 3 for A; n = 6 for B–D). Data are presented as mean ± SEM (n = 3 for A; n = 5 for B; n = 6 for C, D). Statistical significance was determined by an unpaired two-tailed Student's t test for (A) and two-way ANOVA with Tukey's multiple comparisons test for (B–D). * = $P < 0.05$.

Figure S5. ZAKα expression and downstream MAPK signaling in inflammatory macrophages. (A-C) Western blot analysis of phosphorylated JNK (p-JNK) and phosphorylated p38 (p-p38) in WT and ΔGCN2 BMDMs treated with LPS+IFNγ over time, assessing downstream MAPK activation (n=2). (D, E) Phos-tag immunoblot analysis of ZAKα in BMDMs stimulated with LPS+IFNγ (6 h and 24 h) or ANS (2 μM, 15 min). Two independent ZAKα antibodies were used to validate band identity. No phosphorylation-dependent mobility shift of ZAKα was detected under these conditions. (F, G) ZAKα expression after 24h of LPS+IFNγ stimulation compared with non-treated controls, with or without pharmacological inhibition of ZAKα, indicating that ZAKα inhibition preserves its expression during pro-inflammatory stimulation. Data are presented as mean ± SEM (n = 3). Statistical significance was determined by two-way ANOVA with Tukey’s multiple comparisons test for (B, C) and one-way ANOVA with Tukey’s multiple comparisons test for (G). * = $P < 0.05$.

Table 1. Antibodies used in western blot experiments.

Antibody	Provider	Cat. Number	RRID	Blocking buffer	Dilution
Vinculin (E1E9V) XP®	CST	13901S	AB_2728768	TBS-T 3% milk	1:1000
GCN2	CST	3302S	AB_2277617	TBS-T 3% milk	1:1000
p-GCN2 (T899)	Abcam	ab75836	AB_1310260	TBS-T 3% milk	1:1000
p-eIF2alpha (S51) (119A11)	CST	3597S	AB_390740	TBS-T 1% BSA	1:1000
eIF2alpha (D7D3) XP(R)	CST	5324S	AB_10692650	TBS-T 3% milk	1:1000
p-4E-BP1 (T37/46) (236B4)	CST	2855S	AB_560835	TBS-T 3% milk	1:1000
4E-BP1 (53H11)	CST	9644S	AB_2097841	TBS-T 3% milk	1:1000
p-p38	CST	9211S	AB_331641	TBS-T 3% milk	1:1000
p38	CST	9212S	AB_330713	TBS-T 3% milk	1:1000
p-JNK	CST	9251S	AB_331659	TBS-T 3% milk	1:1000
JNK	CST	9252T	AB_2250373	TBS-T 3% milk	1:1000
α-tubulin	CST	2125S	AB_2619646	TBS-T 3% milk	1:1000
ZAKα	Proteintech	28761-1-AP	AB_2918199	TBS-T 3% milk	1:1000
ZAKα	Fortis Life Science	A301-993A	AB_1576612	TBS-T 3% milk	1:1000
Puromycin	Sigma-Aldrich	MABE343	AB_2566826	TBS-T 3% milk	1:10000
RPS10	Abcam	ab151550	AB_2714147	TBS-T 5% BSA	1:1000
GAPDH	Proteintech	60004-1-Ig	AB_2107436	TBS-T 5% BSA	1:10000

Table 2. Primers used in qPCR analyses.

Primers	Sequences
18S-F	ctcaacacgggaaacctcac
18S-R	cgctccaccaactaagaacg
Il1b-F	ggcagctacctgtgtctttccc
Il1b-R	atatgggtccgacagcacgag
Il6-F	ccccaatttccaatgctctcc
Il6-R	cgcactaggtttgccgagta
Tnf-F	atggcctccctctcatcagt
Tnf-R	tttgctacgacgtgggctac
Znf598-F	gaaccggcagattgaccttc
Znf598-R	tacctctccacctcctcgta

Table 3. shRNA sequences used in lentiviral gene silencing.

shRNA	Sequences
shGFP-F	ccgggcaagctgaccctgaagttcactcgagtgaacttcagggtcagcttgctttttg
shGFP-R	aattcaaaaagcaagctgaccctgaagttcactcgagtgaacttcagggtcagcttgc
shZNF598-F	ccggccagtaaccatcgttgtcaatctcgagattgacaacgatggttactggtttttg
shZNF598-R	aattcaaaaaccagtaaccatcgttgtcaatctcgagattgacaacgatggttactgg
